# A nutraceutical intervention for alleviating tired and heavy legs in healthy subjects: a randomized, double-blind, controlled study

**DOI:** 10.1186/s40795-025-01184-1

**Published:** 2025-11-10

**Authors:** Vincenzo Nobile, Enza Cestone, Gloria Roveda

**Affiliations:** 1 R&D Department, Complife Italia S.r.l., San Martino Siccomario, 27028 PV Italy; 2 Clinical Trial Department, Complife Italia S.r.l., San Martino Siccomario, 27028 PV Italy

**Keywords:** Telangiectasias, Symptoms, Diosmin, Clinical trial

## Abstract

**Background:**

Tired and heavy legs are common venous complaints associated with both lifestyle factors and chronic venous insufficiency (CVI).

**Methods:**

This randomized, double-blind, placebo-controlled, study was conducted with 122 healthy subjects experiencing heavy and tired legs and telangiectasia. Participants received one tablet per day of a food supplement containing 570 mg of a formulated flavonoid complex (comprising 80% diosmin and a total flavonoid content of 90%) for 56 days. The control group included subjects with similar baseline characteristics, each receiving one tablet of placebo per day for the same duration. Assessment of leg discomfort, including sensations of heaviness and tiredness, as well as telangiectasia severity, skin color, and blood flow in the affected area, was conducted before (baseline or Day 0) and after 14 (D14), 28 (D28) and 56 (D56) days of supplementation. Additional evaluations included subjective perception of product effectiveness.

**Results:**

By D56, significant improvements were recorded: sensation of heavy legs decreased by 41.7% (*p* < 0.05), while the sensation of tired legs decreased by 45.2% (*p* < 0.01). The number of responders in the active group was approximately twice compared to the placebo group. Participants also reported a positive perception of the product’s efficacy, and the treatment was well-tolerated.

**Conclusions:**

These findings suggest that the tested food supplement provides beneficial effects in alleviating symptoms of heavy and tired legs.

**Trial registration:**

ISRCTN15667863, date of registration14/01/2025.

## Background

Heavy legs are often described as legs that feel weighted, stiff and tired. Although it is not uncommon to occasionally experience sensation of heavy and tired legs, these symptoms may be early signs of vascular peripheral diseases, such as chronic venous insufficiency (CVI) [1, 2] which represents a widespread disorder, whose prevalence is rising constantly [3, 4].

CVI is estimated to affect more than 40% of the U.S. population [5] with lower prevalence in European countries [6–8] and higher in Latin America (up to 68%) [9]. As confirmed by a cross-sectional survey performed by Chiesa and colleagues [2] the most relevant symptoms reported by the 60% of men and 80% of women were heavy and tired legs. These mild venous discomforts typically occur early in life, while more severe symptoms such as pain, a sensation of heat and swollen legs, associated with advanced stages of CVI, may manifest with age [10, 11]. Although the mechanisms underlying the appearance of these symptoms are not fully understood, the progression rate of the sensation of tired and heavy legs is influenced by lifestyle factors [11].

Subjects experiencing sensation of leg heaviness often do not consult their physician until more severe symptoms such as pain, swollen legs, a sensation of heat, varicose veins develop [2]. These early and mild venous signs should be regarded as an “alarm bell” of impaired blood circulation. They indicate the need for preventive measure to avoid the progression of disease and the development of more severe conditions requiring long-term treatment [12]. Subjects with vascular dysfunctions often have major functional changes that can limit daily activities and adversely impact the quality of their life [13].

Heavy legs are sometimes accompanied by the presence of spider veins, also known as telangiectasia, which may appear on the skin and can develop anywhere in the body, although they are primarily localized on the legs. Spider veins are dilated or broken blood vessels near the surface of the skin. Although they are considered a cosmetic issue and are generally harmless, spider veins can affect the self-confidence and social interactions of individuals, with women typically being more concerned than men [14]. In fact, visible veins mainly affect the aesthetic appearance of the skin which can eventually influence individuals’ clothing choices and self-perception, potentially leading to modification in their social activities (e.g. swimming, dancing, or going to the beach) to avoid exposing their legs.

The most common therapeutic approaches for managing the discomfort of tired and heavy legs, as well as telangiectasia, at an early stage, include compression stockings, lifestyle changes, and medications. The efficacy of these treatments and the substantiation of their claims have yet to be conclusively demonstrated. Interventional therapies such as sclerotherapy, radiofrequency, and laser treatment, are often required at advanced stages, when the venous symptoms become more severe. Both sclerotherapy and laser therapy are relatively low-risk procedures with minimal side-effects such as pain at the injection site, bruising, hyperpigmentation, and erythema [15, 16]. However, individuals may be hesitant to undergo medical treatment due to potential side effects and costs, particularly if their expectations are not properly managed. In this context, a food supplement may be more acceptable to affected subjects. Additionally, a clinically validated food supplement could serve as an effective preventive approach to mitigate the progression of more severe CVI symptoms.

Among the venous-supporting compounds commonly used as food supplements, flavonoids and their derivatives have been widely studied [17, 18]. Diosmin is a flavonoid primarily found in Citrus spp. It is known for its anti-inflammatory and antioxidant effects, which help protect blood vessels and endothelial cells, leading to benefits for blood circulation [19]. As with other flavonoids, the efficacy of diosmin is limited by its poor oral absorption [20]. In previous research, the product investigated in the present study (µsmin^®^ Plus, Giellepi S.p.A., Milan, Italy) was shown to have about 4-fold higher relative bioavailability in rats compared to conventional diosmin [21]. These findings were confirmed by a subsequent double-blind, cross-over clinical trial, which revealed a 9.4-fold higher relative bioavailability compared to micronized diosmin in healthy subjects [22]. The better pharmacokinetics profile is reflected in the clinical efficacy of the product, which was effective in improving both quality of life and clinical signs in subjects with CVI classified as C2–C4 according to the Clinical Etiology Anatomy Pathophysiology (CEAP) classification system [23].

Based on this finding, we investigated the efficacy of a food supplement containing this nutraceutical ingredient in alleviating leg discomforts (heavy legs, tired legs, tingling sensation) in healthy subjects at an early stage of CVI – telangiectasias. The efficacy of the product on the appearance of telangiectasias was evaluated as a secondary endpoint.

## Methods

### Trial design

This was a single center, randomized, placebo-controlled trial conducted at San Martino Siccomario (Pavia, Italy) clinical facilities of the Complife Group, from March 2023 to March 2024. The study included a screening visit, a baseline visit (D0) and three follow-up visits (after 14, 28 and 56 days) during a 56-days supplementation period. Eligible subjects were randomized at D0. The primary endpoint was the clinical assessment of leg discomforts (heavy legs, tired legs, and tingling sensation). Secondary endpoints included spider veins severity and appearance (color), blood flow rate, and a self-assessment questionnaire. All study procedures were carried out in accordance with the World Medical Association’s (WMA) Helsinki Declaration and its amendments. The study protocol (prot. no. IT0006501/22 version no. 2) was approved (ref. 2023/01) by the “Independent Ethical Committee for Non-Pharmacological Clinical trials” (Genova, Italy) on 03/02/2023. All subjects signed an informed consent form before any trial-related procedures were initiated. The study adhered to CONSORT guidelines. The trial was registered at ISRCTN registry, number ISRCTN15667863 (10.1186/ISRCTN15667863, accessed on 14 January 2025).

### Trial participants

The sample size was calculated using a two-sided 5% significance level and 80% power, accounting for a 20% variation in the primary endpoint (leg discomforts) due to inter-individual variability and measurement error. Using PASS 11 statistical software (version 11.0.8 for Windows, Microsoft, USA) [24] required sample size of 51 subjects per group was determined. Considering an estimated dropout rate of 20%, the initial sample size for each group was set at 62 participants.

Eligible subjects were healthy men and women, aged between 18 and 60 years, who experienced tired and heavy legs and had telangiectasias. Prior to inclusion, all subjects interested in participating in the trial were screened by a board-certified dermatologist. Only those with tired and heavy legs and telangiectasias classified as C1 according to the CEAP system were included in the trial and scheduled for the baseline visit (D0). Exclusion criteria included a body mass index (BMI) above 30, participation in a similar clinical trial within 30 days (or longer, at investigator’s discretion) prior to the inclusion visit, use of blood flow-enhancing supplements (e.g. citrulline, arginine, beetroot powder) or functional drinks (e.g. pomegranate or beetroot juice) within 30 days before the inclusion visit, pregnancy or breastfeeding, any disorder or therapy (including drugs or food supplements) that could interfere with the study treatment, vascular diseases, diabetes, blood disorders, metabolic, neurological, or orthopedic conditions (including traumas and prior amputation), arthritis, neuropathy, recent vein surgery, or deep or superficial venous thrombosis of the lower limbs within the previous 6 months, smoking ≥ 10 cigarettes/day, and pharmacological treatments interfering with the study outcomes. Additionally, subjects who were regularly exposed to natural or artificial UV light were excluded, as well as those using cosmetic products or topical drugs on the legs.

Two weeks before the study began, the dietary habits of each participant were recorded. Participants were then instructed to maintain their dietary habits and physical activity levels unchanged.

#### Interventions and randomization

1 $$\text{Compliance}\;\text{to}\;\text{treatment}\;\left(\%\right)=\left(\frac{\text{no}.\;\text{of}\;\text{tablets}\;\text{taken}}{\text{no}.\;\text{of}\;\text{tablets}\;\text{to}\;\text{be}\;\text{taken}}\right)\times100$$

Subjects were randomly assigned (1:1 ratio) to either the active or the placebo group using a computed-generated randomization list (PASS 11, version 11.0.8, PASS, LLC. Kaysville, UT, USA) based on the “Efron’s biased coin” algorithm. The active treatment arm received 1 tablet per day of a food supplement containing 570 mg of a proprietary diosmin-based ingredient (µsmin^®^ Plus, Giellepi S.p.A., Milan, Italy) standardized to > 80% diosmin and 90% total flavonoids plus excipients.

The placebo arm received a daily tablet identical in appearance to the active tablet, containing only excipients. The study was double-blind, ensuring neither the subjects nor the team involved in the trial were aware of the active or placebo allocation. Both the investigational product and the placebo were packaged in identical, opaque, white plastic bottles, making their contents indistinguishable from the outside. The tablets (both verum and placebo) had the same size, color, and were odorless, ensuring that neither investigator nor participants could differentiate between them. Each bottle was labeled with a unique alphanumeric code, the meaning of which was unknown to both the investigator and the participant, thereby maintaining blinding throughout the study.

Subjects were instructed to return any unused study product, which was used to assess compliance. The study adhered to maintain separation between the statistician who generated the randomization list, the study staff who enrolled and assigned participants to the intervention, and the investigator who taken the outcomes. The randomization list was securely stored in sequentially numbered, sealed, opaque envelopes. Both the investigator and the participant were blinded to the product assignment.

Compliance to treatment was calculated according to the formula here below:

The compliance threshold was set at ≥ 80%. Participants with compliance less than 80% were excluded from the intention-to-treat (ITT) population due to poor adherence to the treatment regimen.

## Outcomes

Leg discomforts (heavy legs, tired legs, tingling sensation) were self-scored by the subjects using a visual analogue scale (VAS) ranging from 0 (none) to 10 (very strong) [25].

Spider veins severity was assessed by a dermatologist using a scale where 0 indicated absent, 1 very mild, 2 mild, 3 moderate, and 4 severe.

Spider veins color related to the hemoglobin content was measured using a colorimeter (Mexameter^®^ MX18, Courage + Khazaka electronic GmbH, Köln, Germany) in a subpopulation (*N* = 66; *n* = 33 active and *n* = 33 placebo).

Blood flow rate in the spider veins area was measured using a laser doppler (PeriFlux 6000, Perimed Italia Srl, Cuggiono, Milan, Italy).

At the end of the study, subjects were asked to give their opinion on product efficacy by answering a self-assessment questionnaire. Product efficacy was rated on a 5-point scale (strongly disagree or not satisfied at all, disagree or not satisfied, neither disagree nor agree or neither dissatisfied nor satisfied, agree or satisfied, completely agree or very satisfied).

## Statistical methods

Statistical analysis was performed using NCSS 10 (version 10.0.7; NCSS, Kaysville, UT, USA). Data normality was checked using the Shapiro–Wilk W normality test and data shape. The level of statistical significance was reported as follows: * *p* < 0.05, ** *p* < 0.01, and *** *p* < 0.001.

The statistical analysis was carried out on the per protocol (PP) population, which included all randomized subjects who completed the study. Non-parametric data were analyzed using the Wilcoxon test for intragroup comparisons and the Mann-Whitney test was used for the intergroup statistical analysis. Parametric data were analyzed using repeated measures ANOVA (RM-ANOVA), followed by a Tukey-Kramer post hoc test or by Students’ t-test.

## Results

### Study population, tolerability and compliance to treatment

**Fig. 1:**
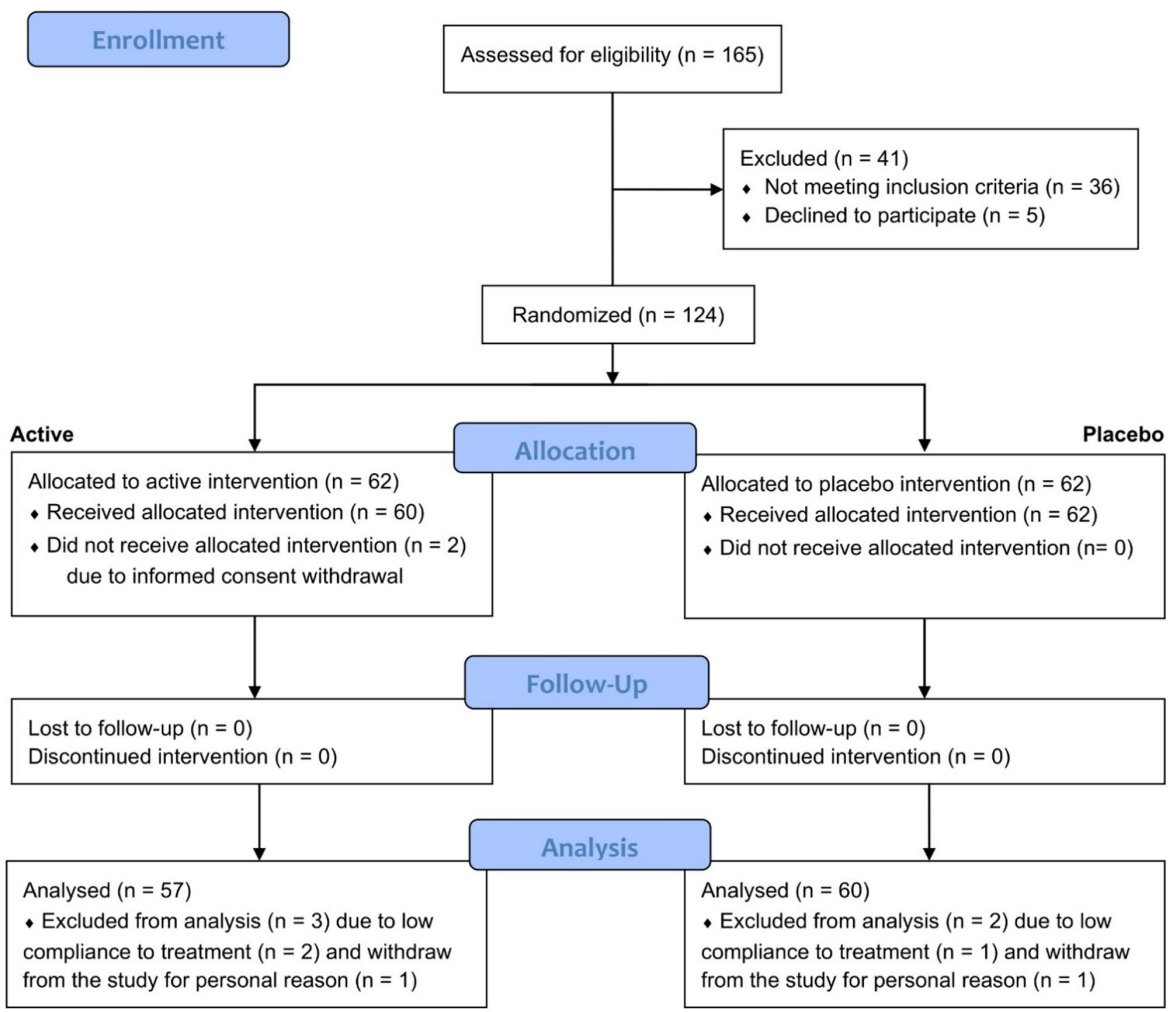
Participants flow chart

A total of 165 subjects were screened for eligibility; of these, 36 did not meet the inclusion criteria, and 5 declined to participate (Fig. 1). The trial then randomized 124 subjects; 60 were allocated to the active group and 62 were allocated to the placebo group. The PP population consisted of 117 subjects. In the active group, 57 subjects completed the study, while in the placebo group, 60 subjects completed the study. The reasons for exclusion from the PP population were one of the following: included withdrawal due to personal reasons (*n* = 2) and intake of < 80% of the test product (*n* = 3).

The mean age (± standard error) was 49.0 ± 1.2 years in the active group and 48.5 ± 1.1 years in the placebo group. Other demographic data at baseline are reported in Table 1. The active and placebo groups were clinically comparable and no significant differences were reordered (*p* > 0.05).

**Table 1:** Demographic data at baseline. Data are means ± SE. In bracket is reported the number of subjects

	Active (*n* = 57)	Placebo (*n* = 60)	Units	*p*-value
Age	49.0 ± 1.2 (min 25; max 60)	48.5 ± 1.1 (min 24; max 60)	Years	> 0.05
CVI according CEAP^a^	C1	C1	Score	> 0.05
Spider veins Severity	2.5 ± 0.1	2.6 ± 0.1	Score	> 0.05
Leg discomforts				> 0.05
Heavy legs	6.0 ± 0.3	5.8 ± 0.3	Score	> 0.05
Tired legs	6.2 ± 0.3	5.6 ± 0.3	Score	> 0.05
Tingling sensation	2.1 ± 0.3	1.7 ± 0.3	Score	> 0.05
Spider veins color(hemoglobin)	330.6 ± 7.5	312.0 ± 10.5	a.u.	> 0.05
Blood flow rate	21.52 ± 1.45	21.71 ± 1.22	p.u.	> 0.05

^a ^Mann-Whitney test

Both the active and the placebo products were well-tolerated, with no adverse effects reported throughout the study period. 

Compliance with the treatment was 97.4% (min. 80.4%, max. 100%) in the active group and 97.2% (min. 80.4%, max. 100%) in the placebo group. Three subjects (2 in the active group and 1 the placebo group) with compliance around 75% were excluded from the ITT population.

**Fig. 2:**
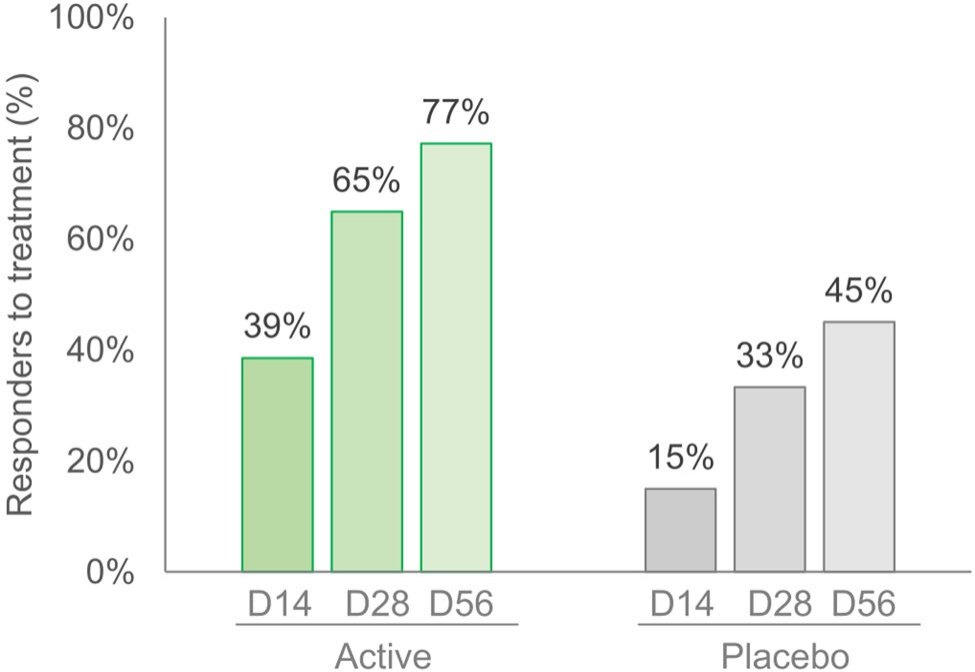
Responders to treatment

The number of subjects who were positively scored as responders (i.e. the number of subjects showing and perceiving an improvement) was approximately twice as high in the active group compared to the placebo group, reaching 77% by the end of the treatment (Fig. 2).

Throughout the entire study period, subjects reported no changes in their dietary habits, physical activity levels, or concurrent pharmacological treatments.

### Leg discomforts

In the actively treated arm, the heavy legs sensation decreased over time by 18.3%, 30.0% and 41.7%, after 14, 28 and 56 days of product intake, respectively (Table 2). A decreasing trend was observed in the placebo group; however, the difference between the active and placebo groups was statistically significant at all checkpoints (*p* < 0.01 at D14 and *p* < 0.05 at D28/D56).

**Table 2:** Leg discomforts (heavy legs, tired legs and tingling). Data are average (± standard error). The intergroup (active vs. placebo) statistical analysis (Mann-Whitney test) is reported as follows

	Active (*n* = 57)				Placebo (*n* = 60)			
	D0	D14	D28	D56	D0	D14	D28	D56
Heavy legs	6.0 ± 0.3	4.9 ± 0.2**	4.2 ± 0.3*	3.5 ± 0.3*	5.8 ± 0.3	5.4 ± 0.3	4.7 ± 0.3	3.9 ± 0.3
Tired legs	6.2 ± 0.3	5.1 ± 0.3**	4.1 ± 0.3**	3.4 ± 0.3*	5.6 ± 0.3	5.3 ± 0.3	4.5 ± 0.3	3.8 ± 0.3
Tingling	2.1 ± 0.3	1.3 ± 0.3	0.8 ± 0.2	0.4 ± 0.2	1.7 ± 0.3	1.2 ± 0.2	0.8 ± 0.2	0.7 ± 0.2

* *p* < 0.05 and ** *p* < 0.01

The tired legs parameter showed improvement starting from D14 (− 17.7%) and was further reduced by 33.9% at D28 and 45.2% at D56 (Table 2). A decreasing trend was recorded in the placebo group as well, but the difference between the active and placebo groups was statistically significant at all checkpoints (*p* < 0.01 at D14/D28 and *p* < 0.05 at D56).

Although there was a trend toward a decrease, the change in tingling sensation in the active group was not statistically different compared to the variation observed in the placebo group.

### Clinical assessment of telangiectasias severity

**Fig. 3:**
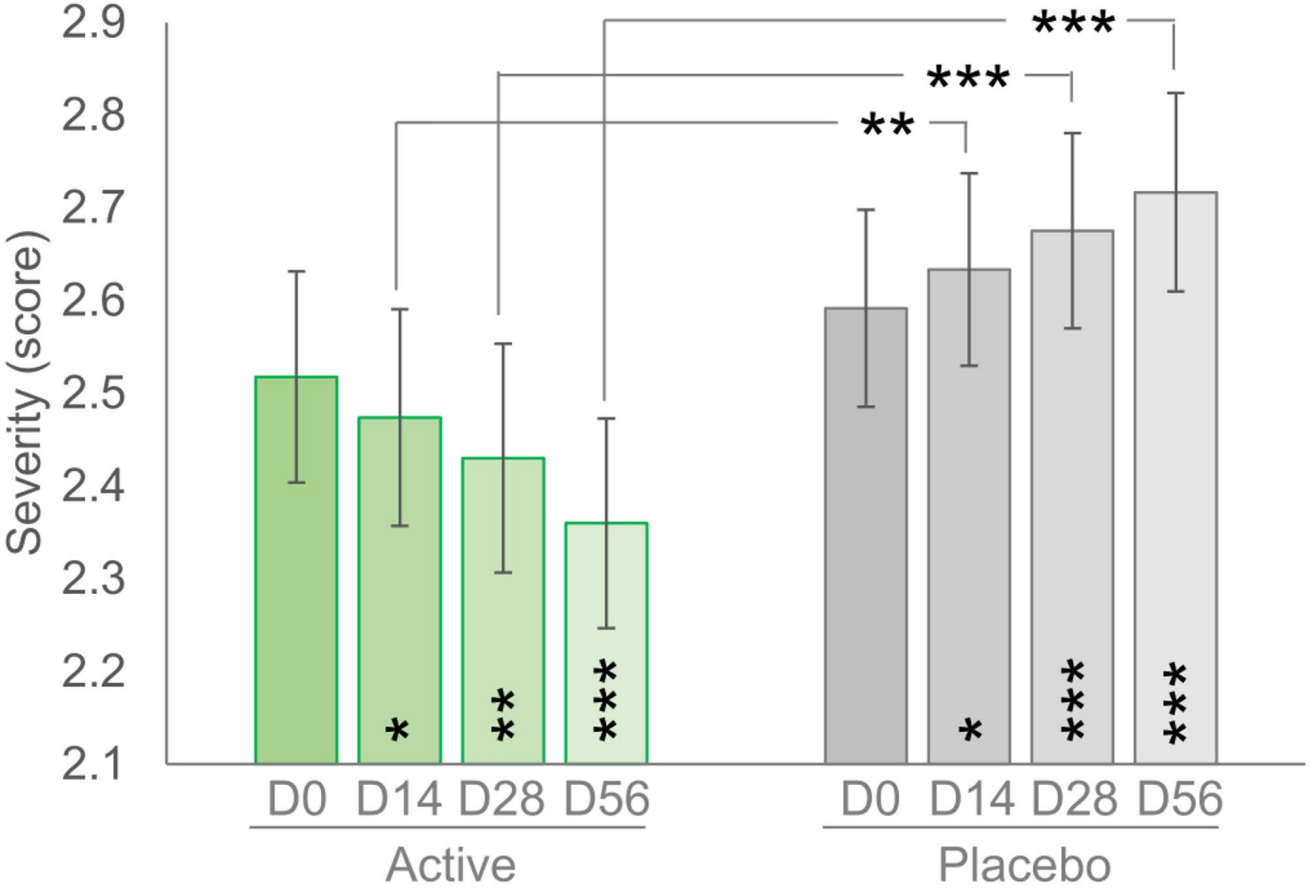
Spider veins severity. Data are average (± standard error). Above the bars is reported the intergroup (active vs. placebo, Wilcoxon test) statistical analysis while inside the bars is reported the intragroup (vs. baseline, Mann-Whitney test) statistical analysis. * *p* < 0.05, ** *p* < 0.01, and *** *p* < 0.001

In the actively treated arm, the telangiectasias severity score, as assessed by the dermatologist, decreased from mild-to-moderate (2.5 ± 0.1) to mild (2.4 ± 0.1) after 56 days of product use (Fig. 3). In the placebo group, the severity of telangiectasias significantly increased (*p* < 0.05), starting from D14. Differences between active and placebo products were statistically significant at all checkpoints, as follows: *p* < 0.01 at D14 and *p* < 0.001 at D28 and D56. Although the effect size of the parameter variation in the active group was small, it is noteworthy that the active treatment helped counteract the physiological worsening of telangiectasias severity noted in the placebo group.

### Blood flow rate

The blood flow rate remained unchanged in both the active and placebo groups throughout the study period (Table 3).

**Table 3:** Blood flow rate. Data are average (± standard error). No statistically significant difference (RM-ANOVA, followed by a Tukey-Kramer post hoc test) were found between the groups (*p* > 0.05). p.u. Perfusion units

	Active (*n* = 57)				Placebo (*n* = 60)			
	D0	D14	D28	D56	D0	D14	D28	D56
Blood flow (p.u.)	21.52 ± 1.45	20.16 ± 1.14	21.33 ± 1.46	18.96 ± 1.03	21.71 ± 1.22	21.94 ± 1.51	19.39 ± 0.83	19.07 ± 1.08

### Measurement of telangiectasias color

**Fig. 4:**
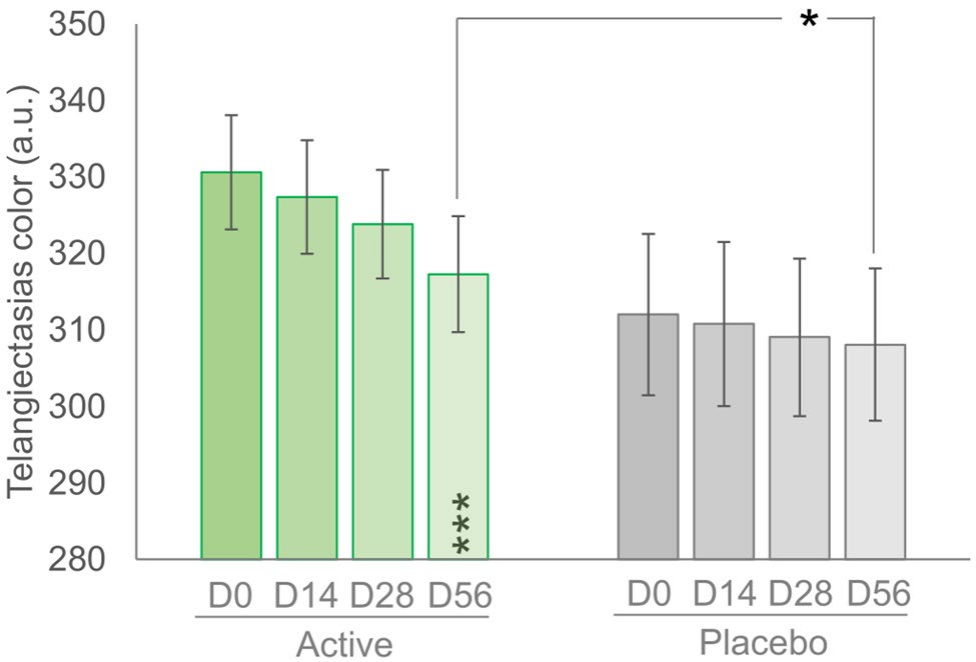
Telangiectasias color. Data are average (± standard error). Above the bars is reported the intergroup (active vs. placebo, Student’s t-test) statistical analysis while inside the bars is reported the intragroup (vs. baseline, RM-ANOVA, followed by a Tukey-Kramer post hoc test) statistical analysis. * *p* < 0.05 and *** *p* < 0.001

In the active group, the telangiectasias color related to the hemoglobin content significantly decreased by 3.9% after 56 days of product use (*p* < 0.001), while it remained unchanged in the placebo group (Fig. 4). A reduction in this parameter was observed in 70% of the subjects. Differences between the active and placebo groups were statistically significant (*p* < 0.05).

#### Self-assessment questionnaire

**Fig. 5:**
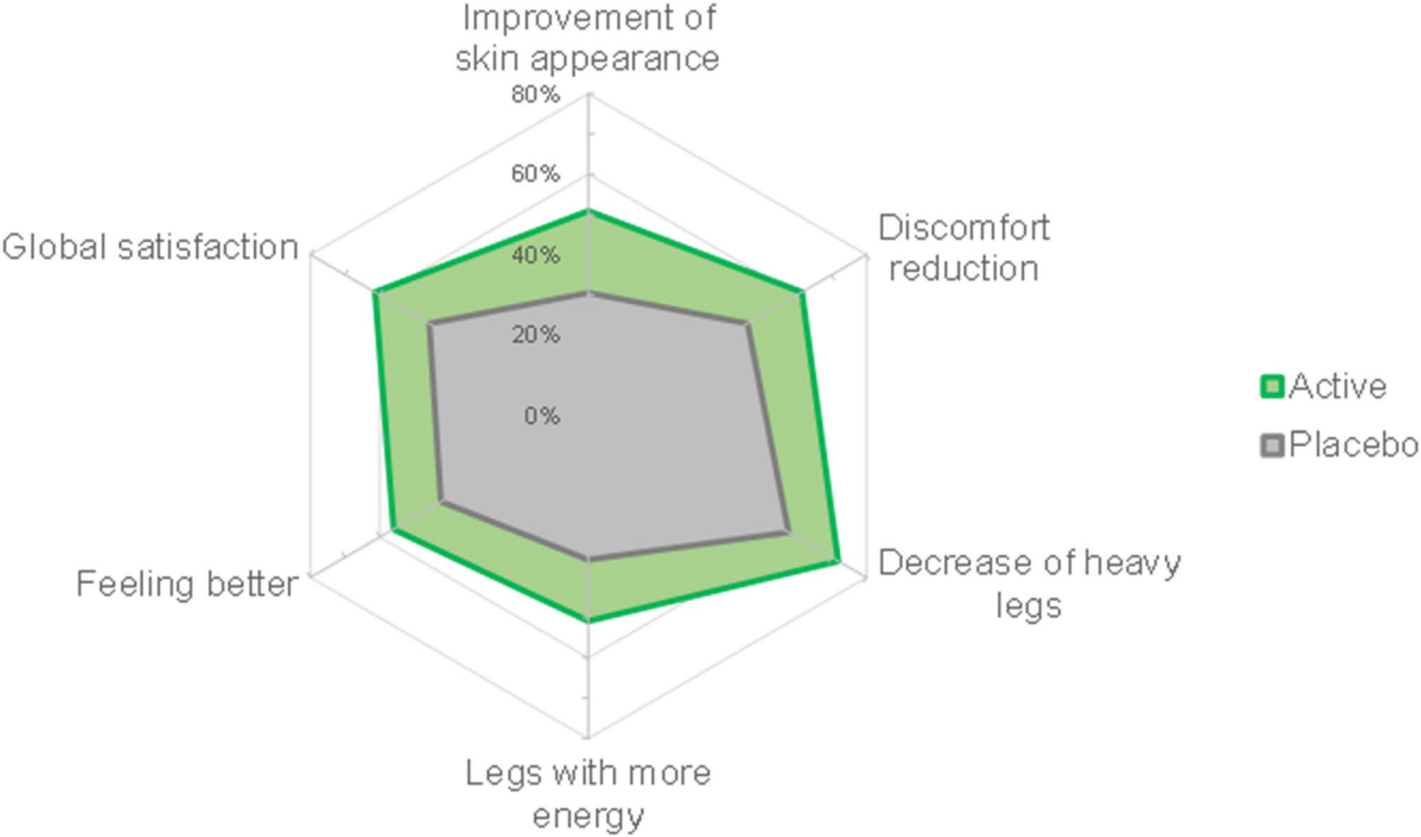
Self-assessment questionnaire. The graph reports the percentage of positive answers (i.e., “completely agree” and “agree”, or “very satisfied” and “satisfied”)

The self-assessment questionnaire results were most favorable for the active product. Participants in the active group reported perceived improvements in skin appearance (50.9%), reduction of the overall discomforts (61.4%), decrease of the heavy legs (71.9%), increase in leg energy (50.9%), and an overall better feeling (56.1%). Additionally, 61.4% of the subjects using the active product were globally satisfied with the obtained results. In contrast, the percentage of subjects reporting improvements in the placebo arm was lower, ranging from 30.5% to 57.6%, compared to the active treatment arm (Fig. 5).

## Discussion

Tired and heavy legs are widespread symptoms, with a prevalence that is steadily increasing worldwide [3]. They are exacerbated by prolonged standing, sitting, or a sedentary lifestyle [26, 27]. However, these discomforts are frequently overlooked, and patients typically seek medical consultation only when CVI progresses to more severe stages [2].

Although tired and heavy legs are an early and mild symptoms associated with higher CEAP stages, scientific studies investigating this condition and its treatment remain scarce. Conversely, numerous products on the market claim to alleviate this condition. However, in most cases, these claims lack sufficient scientific validation [2].

While diosmin, a flavonoid commonly used in the management of venous disorders, shows promise in improving symptoms of CVI and may offer some benefits for subjects with C1 telangiectasia, direct evidence in this specific population is still lacking. The present study provides new insights into the potential relief of leg discomforts in subjects classified as C1 according to the CEAP classification. C1-classified subjects show early manifestation of a more complex condition (CVI) without detectable anatomical or pathophysiological anomalies. Unlike the more severe CEAP classes (>C2), this category is not adequately represented in the international scientific literature, and clinical data remain scarce.

The results of this study demonstrated the efficacy of the tested flavonoid complex in improving sensations of heavy legs (− 41.7%) and tired legs (− 45.2%). Additionally, although not statistically significant, there was a trend toward a reduction in tingling sensations. On the other hand, the variation of the discomforts within the placebo group was not statistically significant and appeared to be influenced by individual susceptibility. These findings suggest that the investigated dietary supplement may have beneficial effects on venous function, potentially by enhancing microcirculation, reducing venous pressure, or exerting anti-inflammatory effects, which are all well-established actions of diosmin [17, 19]. The lack of improvement in tingling sensations found in our trial, might indicate that this sensation is less related to venous insufficiency at this early stage (C1 classification) and could be influenced by other factors such as nerve function or more advanced venous disease. Moreover, tingling (also known as paresthesia) was non-significantly reduced following the supplementation with micronized purified flavonoid fraction (MPFF) compared with placebo when assessed as a continuous variable in a cohort of 150 participants with CVI [28]. These findings provide the additional evidence that diosmin may work preferably on other signs of CVI mainly legs heaviness and tiredness.

Efficacy in the actively treated group was also observed in terms of improved skin appearance in areas affected by spider veins and a better overall sensation. The improvement in the measured parameters was not associated with changes in blood flow rate in the affected area. However, a variation in the color of the telangiectasia, related to the hemoglobin content of the skin, was detected using a colorimeter and a significant improvement was observed at D56 compared to both baseline (*p* < 0.001) and placebo (*p* < 0.05). This behavior can be explained by a reduction in blood stasis, accompanied by very small changes in blood flow rate that are undetectable by the laser doppler techniques, as well as individual variability in response.

The lack of a significant change in blood flow rate despite improvements in other parameters may be attributed to the specific mode of action of diosmin. As a venoactive agent, diosmin primarily enhances venous tone, reduces capillary permeability, and improves lymphatic drainage rather than directly increasing blood flow. Additionally, the methodology used to assess blood flow (laser doppler) mainly detects microcirculatory changes, which may not fully capture the hemodynamic improvements occurring at the deeper venous level. Further investigations employing complementary techniques could provide a more comprehensive understanding of these mechanisms.

Diosmin is thought to enhance venous tone and reduce capillary permeability, which could theoretically benefit subjects with telangiectasia or spider veins (C1) by reducing the visible appearance of these veins. Although direct evidence for its effectiveness specifically for telangiectasia is limited, several studies have shown that diosmin can reduce skin redness and erythema, symptoms relevant to those with spider veins [28]. In our trial, a moderate improvement in the color of telangiectasia (− 3.9%) was observed in 70% of the subjects, while a visually noticeable improvement in appearance was seen in a smaller proportion. Although the effect size of the parameter variation in the active group was modest, it is noteworthy that the active treatment counteracted the physiological worsening of the telangiectasia severity observed in the placebo group. These data may suggest a preventive effect of the ingredient, which may help prevent the progression of the symptoms to more severe stages. However, a confirmatory study is needed to strengthen this conclusion. Our results are consistent with those reported by Greco and Bissacco in a previous clinical trial, where subjects were supplemented with a food supplement containing an equivalent amount of the same nutraceutical ingredient investigated in the present study. The supplement effectively relieved venous symptoms, including pain, heaviness, and fatigue, in individuals with CVI classified as C0–C1 [29].

In a large multicenter, observational study, 70 physicians recruited 1,150 CEAP C1 subjects undergoing sclerotherapy for the treatment of telangiectasia [30]. Of these, 905 subjects (79%) were treated with 1,000 mg/day of micronized purified flavonoid fraction (MPFF) for 6 weeks, starting 2 weeks prior to sclerotherapy. These subjects experienced greater relief from symptoms, such as leg heaviness, pain and swelling, compared to those who underwent sclerotherapy alone. This suggests a rationale for the adjunctive use of venoactive compounds, such as diosmin, during phlebosclerosing interventions.

Subjects with C1 classification often experience emotional distress due to the appearance of these veins and subsequent changes in skin color. The visibility of spider veins, particularly in socially exposed areas such as legs, can result in decreased self-esteem, body image concerns, and a reluctance to engage in social or physical activities, such as swimming or wearing certain types of clothing. Additionally, symptoms such as leg heaviness and fatigue may negatively affect daily activities, further limiting mobility and participation in routine tasks. This combination of physical discomfort and emotional burden can exacerbate feelings of embarrassment or self-consciousness, potentially leading to social withdrawal and a diminished quality of life [31].

Moreover, the chronic and progressive nature of venous insufficiency and telangiectasia can contribute to anxiety and a sense of helplessness, as patients may be concerned about the worsening of their condition or the potential ineffectiveness of treatments. Although spider veins are often viewed primarily as a cosmetic issue, their impact on mental health and overall well-being should not be underestimated. Therefore, early management of telangiectasia in C1 subjects is crucial not only for delaying the onset of more severe symptoms and preventing progression to higher CEAP classes but also for maintaining optimal legs health.

To the best of Author’s knowledge, this is one of the very few studies conducted in the C0-C1 population. This condition is common yet underestimated, leading to functional limitations and a reduced quality of life. Therefore, early intervention is crucial, and the findings of this study highlight the effectiveness of the investigational product in alleviating legs discomfort, particularly heaviness and fatigue. Moreover, the supplement was well tolerated, with no side effects reported throughout the study, confirming its safety and tolerability. However, this study has some limitations. No follow-up was planned, so no data was collected after the study’s conclusion. Additionally, all recruited subjects were healthy individuals aged 18–60 years with a BMI < 30, which may limit the generalizability of the findings. However, these homogeneous characteristics were intentionally selected to minimize bias and confounding factors that could affect the assessed parameters. Another limitation is that the primary outcome was evaluated using self-reported questionnaires. Nevertheless, statistically significant differences were observed between the two treatment groups and the sample size was robust, having been determined through a preliminary statistical calculation.

## Conclusion

The results of this study confirm the vasoactive efficacy of the tested diosmin formulation in healthy subjects classified as C1 according to the CEAP classification of CVI. The improvement in leg discomfort, along with the favorable safety profile of the treatment, highlights the suitability of the tested formulation for individuals for whom standard treatments (e.g., compression stockings) are contraindicated or who prefer to avoid certain interventions (e.g., sclerotherapy, radiofrequency, and laser) due to undesirable side effects, such as ecchymosis and hyperpigmentation, or because these interventions are considered invasive (e.g., intradermal puncture) or costly. Given these findings, the study suggests that the diosmin-based supplement may provide measurable benefits in reducing leg discomfort and improving the skin color associated with telangiectasia. However, the lack of impact on tingling sensation and the use of a non-validated efficacy scale may limit the conclusion drawable from this trial. Further research with a larger sample size and extended follow-up is necessary to more accurately establish the efficacy of diosmin in this context and to confirm our preliminary findings regarding discomfort relief and cosmetic outcomes, thereby providing a more comprehensive evaluation of its therapeutic potential for this condition.

## Data Availability

The raw data supporting the conclusions of this article will be made available by the authors on request. The data are not publicly available since they are the property of the sponsor of the study (Giellepi S.p.A. Milan, Italy).
